# Treatment of hepatitis C in special populations

**DOI:** 10.1007/s00535-017-1427-x

**Published:** 2018-01-03

**Authors:** Goki Suda, Koji Ogawa, Kenichi Morikawa, Naoya Sakamoto

**Affiliations:** Department of Gastroenterology and Hepatology, Graduate School of Medicine, Hokkaido University, North 15, West 7, Kita-ku, Sapporo, Hokkaido 060-8638 Japan

**Keywords:** Direct-acting antiviral, HCV, Hemodialysis, HIV, Liver transplantation

## Abstract

Hepatitis C virus (HCV) infection is one of the primary causes of liver cirrhosis and hepatocellular carcinoma. In hemodialysis patients, the rate of HCV infection is high and is moreover associated with a poor prognosis. In liver transplantation patients with HCV infection, recurrent HCV infection is universal, and re-infected HCV causes rapid progression of liver fibrosis and graft loss. Additionally, in patients with HCV and human immunodeficiency virus (HIV) co-infection, liver fibrosis progresses rapidly. Thus, there is an acute need for prompt treatment of HCV infection in these special populations (i.e., hemodialysis, liver transplantation, HIV co-infection). However, until recently, the standard anti-HCV treatment involved the use of interferon-based therapy. In these special populations, interferon-based therapies could not achieve a high rate of sustained viral response and moreover were associated with a higher rate of adverse events. With the development of novel direct-acting antivirals (DAAs), the landscape of anti-HCV therapy for special populations has changed dramatically. Indeed, in special populations treated with interferon-free DAAs, the sustained viral response rate was above 90%, with a lower incidence and severity of adverse events.

## Introduction

Worldwide, it is estimated that 130–150 million people are currently infected with hepatitis C virus (HCV). HCV infection is one of the primary causes of liver cirrhosis and hepatocellular carcinoma [1–3]. HCV infection is also a common cause of liver transplantation (LT) [4], and can lead to renal dysfunction [5, 6]. The rate of HCV infection is high in patients with end-stage renal dysfunction, especially in those on hemodialysis, as well as in patients infected with the human immunodeficiency virus (HIV) [7–9] [10, 11]. Importantly, the prognosis of hemodialysis patients with HCV infection is significantly worse than that of patients without HCV infection [12–14]. Thus, the Kidney Disease: Improving Global Outcomes (KDIGO) guidelines highly recommend anti-HCV therapy for hemodialysis patients with HCV infection, provided that their life prognosis is favorable [6]. In liver-transplanted patients with HCV infection, recurrent HCV infection of the transplanted liver is universal [15], and re-infected HCV causes rapid progression of liver fibrosis [16, 17]. In patients with HCV/HIV co-infection, liver fibrosis progresses more rapidly than in patients with HCV monoinfection, and many patients develop liver cirrhosis [18, 19]. Due to progress in antiretroviral therapy (ART), the mortality rate associated with acquired immunodeficiency syndrome (AIDS) has been decreasing [20], and liver-disease-related death is currently the second most common cause of non-AIDS death in patients with HIV/HCV co-infection. Thus, there is an urgent need for prompt treatment of HCV infection in patients with HIV/HCV co-infection, patients with recurrent HCV infection after LT, and patients undergoing dialysis, whom we shall henceforth refer to as “special populations.” Until recently, the standard anti-HCV therapy involved the use of interferon (IFN)-based agents. However, IFN-based therapy for HCV could not achieve a high rate of sustained viral response (SVR) even in patients without complications, and provided even poorer SVR rates in special populations. In addition, treatment-induced adverse events (AEs) were more frequent in special populations than in HCV-infected patients without complications; these included rejection reactions against the liver graft in LT patients with recurrent HCV infection as well as severe anemia in hemodialysis patients and in HIV/HCV-co-infected patients treated with a certain type of anti-HIV regent. [21–23]. Therefore, safe and effective anti-HCV therapies are critical in these special populations.

A novel class of drugs referred to as direct-acting antivirals (DAAs) has recently been developed. DAAs directly target certain viral proteins such as HCV nonstructural (NS) proteins (HCV NS3, NS5A, and NS5B). Several clinical trials and real-world data have shown that IFN-free DAA-based therapies provide significantly better safety and therapeutic efficacy [24–30]. Initially, the majority of DAA trials excluded special populations, and thus the efficacy and safety of IFN-free DAA therapy in special populations remained unclear. However, several recent studies have shown that combination therapy with IFN-free DAAs offers high efficacy and safety even in special populations [31–34] [35–37] [38, 39]. This recent evidence has revolutionized anti-HCV treatment and provided adequate therapeutic strategies for dialysis patients, patients with HIV co-infection, and patients with recurrent HCV infection after LT (Table 1). On the other hand, drug–drug interactions remain a concern in all patients with comorbidities, and further investigation is warranted to clarify potential interactions between DAAs and immunosuppressive drugs (in patients with LT) or ARTs (in patients with HIV co-infection). Furthermore, careful attention should be paid to DAA elimination, especially in HCV-infected patients with severe renal dysfunction.

**Table 1:** Safety and efficacy comparison between IFN-based therapy and IFN-free DAA therapy in special populations

Indicator/population	IFN-based therapy	IFN-free DAA therapy
SVR rate %		
Hemodialysis	40–41% [21, 49]	90–100% [31, 32, 47, 58, 60, 75]
HIV/HCV co-infection	27–40% [79–81]	78–98% [35, 36, 84, 85, 87, 88, 90, 107]
Liver transplantation	8–50% [95]	70–98% [38, 39, 85, 99, 101–103, 105, 106]
Treatment discontinuation rate		
Hemodialysis	14–16% [21, 49]	0–5% [31, 32, 47, 58, 60, 75]
HIV/HCV co-infection	12–39% [79–81]	0–3% [35, 36, 84, 85, 87, 88, 90, 107]
Liver transplantation	27.6% [95]	2–18% [38, 39, 85, 99, 101–103, 105, 106]

*IFN* interferon, *DAA* direct-acting antiviral, *HIV* human immunodeficiency virus, *HCV* hepatitis C virus

In this review, we discuss the evolution, current state, and remaining concerns in anti-HCV therapy for special populations such as patients on hemodialysis, patients with HIV co-infection, and patients with recurrent HCV infection after LT.

## HCV infection in patients receiving hemodialysis

Patients with end-stage renal dysfunction (including those on hemodialysis) are more susceptible to HCV infection [7–9, 40, 41]. Moreover, HCV infection itself causes renal dysfunction [5], including membrane-proliferative glomerulonephritis via mixed cryoglobulinemia [42], and increases the risk of developing end-stage renal disease [43]. Importantly, HCV infection is a predictor of poor prognosis in hemodialysis patients, although it may worsen prognosis even in patients with normal renal function [44, 45]. In addition, HCV infection reduces the rate and length of survival of renal allografts [14]. Thus, various guidelines highly recommend anti-HCV therapy in HCV-infected dialysis patients, provided that their life prognosis is favorable [6, 46]. Treatment strategies in HCV-infected dialysis patients have shifted from IFN-based to IFN-free DAA therapies, based on recent evidence from clinical trials and studies in the clinical setting [31, 32, 47] (Table 2).

**Table 2:** Outcomes of combination therapies with IFN-free DAAs in patients who have severe renal dysfunction and/or are receiving HD

Treatment regimen (treatment duration) study	Type of study	Patient population	HCV GT	Sample size	SVR rate (%)	SAE (%)	Treatment discontinuation rate (%)	Comments
Grazoprevir/elbasvir (12 weeks)								AEs were comparable between intervention and placebo groups
C-SURFER Roth et al. [47]	CT	CKD 4/5 including HD	GT1	122 (HD: 92)	94.2% (115/122)	14.4% (16/111)	4.1% (5/122)^a^	
C-SURFER Bruchfeld et al. [58]	CT	CKD 4/5 including HD	GT1	102	95.1% (97/102)	12.7% (13/102)	2.9% (3/102)	
PTV/RTV/OBV and DSV ± RBV (12 weeks)								Should be monitored for DDIs
RUBY-1 Pockros et al. [31]	CT	CKD 4/5 including HD	GT1	20 (CKD5/HD: 14)	90% (18/20)	20% (4/20)	0	
PTV/RTV/OBV (12 weeks)								
Atsukawa et al. [60]	RW	HD	GT1b	31	96.8% (30/31)	3.2% (1/31)	3.2% (1/31)	
DCV/ASV (24 weeks)								24-Week treatment regimen SVR rate was not high in patients infected with GT1a
Suda et al. [33]	RW	HD	GT1	21	95.2% (20/21)	5% (1/21)	5% (1/21)	
Toyoda et al. [66]	RW	HD	GT1b	28	100% (28/28)		3.6% (1/28)	
Kawakami et al. [67]	RW	HD	GT1	18	100% (18/18)	6% (1/18)	0%	
Miyazaki et al. [68]	RW	HD	GT1b	10	100% (10/10)	20% (2/10)	0%	
Suda et al. [32]	RW	HD	GT1	123	95.9% (118/123)		3.3% (4/123)	
Glecaprevir/pibrentasvir (12 weeks)								Pan-genotypic activity
Gane et al. [75]	CT	CKD4/5 including HD	GT1-6	104 (HD: 85)	98% (102/104)	24% (25/104)	4% (4/104)	
SOF-based therapy SOF/PEG-IFN/RBV, SOF/RBV, SOF/SMV, SOF/SMV/RBV								SOF is still not recommended for patients with severe renal dysfunction
Saxena et al. [71]	RW	eGFR < 45 mL/min/1.73 m^2^	GT1-6	73 (HD: 5)	83% (53/64)	22% (16/73)	7% (5/73)	
		eGFR < 30 mL/min/1.73 m^2^	GT1-3	17 (HD: 5)	88% (15/17)	18% (3/17)	6% (1/17)	
SOF/SMV (12 weeks)								
Nazario et al. [72]	RW	eGFR < 30 mL/min/1.73 m^2^ including HD	GT1	17 (HD: 15)	100% (17/17)	0	0	

*CT* clinical trials, *RW* real-world data, *IFN* interferon, *DAAs* direct-acting antivirals, *PTV* paritaprevir, *OBV* ombitasvir, *DSV* dasabuvir, *SOF* sofosbuvir, *RBV* ribavirin, *RTV* ritonavir, *Peg-IFN* pegylated interferon, *SMV* simeprevir, *DCV* daclatasvir, *ASV* asunaprevir, *eGFR* estimated glomerular filtration rate, *GT* genotype, *SVR* sustained viral response, *SAE* serious adverse event, *HD* hemodialysis, *CKD* chronic kidney disease, *DDI* drug–drug interactions

^a^ All-treatment discontinuation due to unrelated AEs

### IFN-based therapy in hemodialysis patients

Until recently, IFN monotherapy or IFN in combination with ribavirin was the standard treatment strategy in HCV-infected patients regardless of renal function [6]. However, the SVR rate was not high among hemodialysis patients, partially because of a high incidence of AEs that led to treatment discontinuation. Careful monitoring for AEs is required in patients with severe renal dysfunction because both IFN and ribavirin are mainly excreted renally [48], ribavirin cannot be eliminated by dialysis [46, 48], and renal anemia caused by renal dysfunction can be aggravated by IFN- or ribavirin-induced anemia in hemodialysis patients. In fact, in Japan, ribavirin administration is contraindicated in patients with severe renal dysfunction [46, 48].

In their meta-analysis regarding the safety and efficacy of IFN monotherapy in dialysis patients with HCV infection, Gordon et al. [21] reviewed 20 clinical studies and reported an overall estimated SVR rate of 41% (95% confidence interval [95% CI] 33–49%) and an overall treatment discontinuation rate of 26% (95% CI 20–34%). A recent meta-analysis by Fabrizi et al. [49] focused on the safety and efficacy of pegylated (Peg)-IFN monotherapy in HCV-infected patients on chronic hemodialysis. In this analysis, which included 744 patients described in 24 clinical studies, the overall estimated SVR rate was 40% (95% CI 35–46%) and the estimated treatment discontinuation rate was 14% (95% CI 9–20%). Thus, IFN or Peg-IFN monotherapy showed limited efficacy and was associated with a high rate of AEs, which is why such therapies have not been widely adopted in hemodialysis patients.

First-generation protease inhibitors such as telaprevir, boceprevir, and combination therapy with Peg-IFN and ribavirin achieved an SVR rate of 75–85% in patients with normal renal function [50–52]. However, severe AEs were reported, including cutaneous rash [53], anemia, and renal impairment. The few studies that evaluated the therapeutic effectiveness of such agents in HCV-infected patients with renal dysfunction [54–56] reported highly variable SVR rates ranging from 17 to 86%.

### IFN-free DAA combination therapy in hemodialysis patients

IFN-free DAA therapies were widely adopted as anti-HCV treatment strategies for dialysis patients once their safety and high effectiveness had been demonstrated even in HCV-infected patients with severe renal dysfunction, including those receiving hemodialysis. However, the administration of DAA to patients on hemodialysis should be performed with careful consideration of the DAA elimination route. For instance, sofosbuvir, one of the most potent DAAs, is metabolized via the kidney. Drug–drug interactions are another cause for concern, since hemodialysis patients typically have complex prescriptions.

#### Grazoprevir and elbasvir

The HCV NS3 protease inhibitor grazoprevir and the HCV NS5A inhibitor elbasvir are eliminated mostly through the liver, with less than 1% being excreted renally [57]. Pharmacokinetics analysis of elbasvir and grazoprevir showed that the area under the blood concentration–time curve (AUC) was, respectively, 25 and 10% higher in hemodialysis subjects than in subjects with normal renal function [57]. Thus, combination therapy with these DAAs could be administered to patients with severe renal dysfunction, including those receiving hemodialysis.

The phase 3 trial C-SURFER demonstrated the efficacy and safety of grazoprevir and elbasvir combination therapy in patients with HCV genotype (GT) 1 infection and severe renal dysfunction, including those receiving hemodialysis [31]. Among all the patients screened in this trial, 244 with severe renal dysfunction (including 179 hemodialysis patients) were randomly assigned to receive grazoprevir and elbasvir for 12 weeks (*n* = 111; immediate treatment group) or a placebo (*n* = 113; deferred treatment group). Independently from the randomization, 11 patients were administered grazoprevir and elbasvir, and pharmacokinetic evaluations were conducted. Overall, in patients treated with grazoprevir and elbasvir (111 randomized patients plus 11 patients who underwent pharmacokinetic evaluations), the SVR rate at 12 weeks (SVR_12_) was 94.3% (115/122) in the intention-to-treat analysis set and 99% (115/116) in the modified full analysis set. No patients in the grazoprevir and elbasvir therapy group discontinued due to AEs. In fact, the rate of AEs did not differ between the treatment group and the placebo group. Additionally, no change in the mean estimated glomerular filtration rate (eGFR) or creatinine levels was observed. Quite recently, additional results of the C-SURFER study regarding treatment outcomes of the deferred group were reported. Overall, the SVR rate in the modified full analysis set was 98% (97/99), and the safety profile was similar to that of the immediate treatment group [58]. These reports clearly indicated that grazoprevir and elbasvir treatment for 12 weeks was safe and highly effective, even in patients with HCV GT1 infection and stage 4–5 chronic kidney disease (CKD).

#### Paritaprevir/ritonavir and ombitasvir with or without dasabuvir

The second-generation NS3 protease inhibitor paritaprevir is metabolized mainly through the liver, with AUC values reported to be 45% higher in subjects with severe renal dysfunction than in control subjects [59]. Ritonavir, which is administered to inhibit cytochrome P450 3A4 (CYP3A4), thus enhancing the effect of paritaprevir, was reported to have AUC values 114% higher in subjects with severe renal dysfunction than in control subjects. The HCV NS5A inhibitor ombitasvir is excreted via the biliary route, and its AUC values were similar in subjects with severe renal dysfunction and in those with normal renal function. The non-nucleoside NS5B polymerase inhibitor dasabuvir is also mainly metabolized through the liver; it has AUC values that are 50% higher in subjects with severe renal dysfunction than in control subjects [34]. Thus, combination therapy with these DAAs is not contraindicated in patients with severe renal dysfunction. Indeed, several recent studies have showed the safety and efficacy of combination therapy with ombitasvir, paritaprevir, and ritonavir ± dasabuvir in HCV-infected patients with severe renal dysfunction, including those receiving hemodialysis [31, 60]. However, in this regimen, special attention should be paid to drug–drug interactions, because ritonavir strongly inhibits CYP3A4.

The RUBY-I study showed the safety and efficacy of combination therapy with paritaprevir/ritonavir, ombitasvir, and dasabuvir ± ribavirin in patients with stage G4 or G5 CKD [31]. Twenty treatment-naïve, noncirrhotic patients with HCV GT1 infection and severe renal dysfunction were enrolled in this prospective study. Of those, 14 patients had CKD stage 5 or received hemodialysis. Patients with HCV GT1a infection (*n* = 13) were treated with this combination therapy plus ribavirin, while patients with HCV GT1b infection (*n* = 7) were treated without ribavirin. The overall SVR_12_ rate was 90% (95% CI 69.9–97.2%, 18/20). All 20 patients completed the treatment. Most AEs were mild or moderate, and no renal AEs were reported.

Atsukawa et al. retrospectively analyzed the safety and efficacy of paritaprevir/ritonavir and ombitasvir in 31 hemodialysis patients with HCV GT1b infection [60]. The overall SVR_12_ rate was 96.8% (30/31). Eleven patients (35.5%) experienced AEs. One patient discontinued this combination therapy due to AEs and experienced virological relapse. Concomitant drugs were discontinued or modified in 41.3% of the enrolled patients. Muñoz-Gómez et al. reported the safety and efficacy of ombitasvir/paritaprevir/ritonavir ± dasabuvir ± ribavirin in 46 patients with CKD stage 4/5 and HCV GT1–4 infection [61]. Of the 46 patients included in this multicenter retrospective study, 41.3% had to discontinue or modify their prescription of concomitant drugs. The overall SVR_12_ rate (intention-to-treat analysis set) was 95.7%. Two patients (4.3%) discontinued this treatment due to AEs unrelated to antiviral therapy.

#### Asunaprevir and daclatasvir

The combination therapy with the NS5A inhibitor daclatasvir and the second-generation NS3 protease inhibitor asunaprevir was the first IFN-free DAA combination therapy to be approved in Japan. Asunaprevir is metabolized by CYP3A and eliminated mostly in the feces [62]. The pharmacokinetics of asunaprevir indicated that the peak serum concentration is 28.6% higher and the AUC is 10.1% lower in dialysis subjects than in subjects with normal renal function. For daclatasvir, the AUC is 26.9% higher in dialysis subjects than in subjects with normal function [63]. Thus, combination therapy with daclatasvir/asunaprevir is not contraindicated in HCV-infected patients with severe renal dysfunction, including those receiving dialysis. In phase 3 trials enrolling patients with HCV GT1b infection and without severe renal dysfunction, a 24-week daclatasvir/asunaprevir combination therapy had a high SVR rate (84.2–84.6%) [27, 64], with grade 3 AEs related to renal function being observed in only 1 of 222 patients (0.5%) [27]. Our study of real-world data confirmed that this therapy does not detrimentally affect renal function in nonhemodialysis patients [65].

In the same study, which also enrolled 21 hemodialysis patients with HCV GT1 infection, we reported the efficacy and safety of daclatasvir/asunaprevir combination therapy [65], with an overall SVR_12_ rate of 95.5% (20/21). All 3 hemodialysis patients with the NS5A resistance-associated variant Y93H achieved SVR_12_. One patient (4.8%) discontinued treatment due to AEs. Toyoda et al. [66] also reported the efficacy and safety of daclatasvir/asunaprevir combination therapy for HCV GT1b-infected dialysis patients (overall SVR_12_ rate, 100%; 28/28), and treatment-related AEs were similar to those noted among patients with normal renal function. Other reports [67, 68] confirmed the high efficacy of this combination therapy in hemodialysis patients, but included a relatively limited number of patients. We thus analyzed the efficacy and safety of this combination therapy in a nationwide retrospective study involving 123 hemodialysis patients with HCV GT1 infection. The overall SVR_12_ rate was 95.9% (118/123), and 94.4% (17/18) of hemodialysis patients with NS5A resistance-associated variants achieved SVR_12_. Advanced fibrosis and the presence of the interleukin-28B non-TT GT at rs8099917 were significantly associated with non-SVR. Only 3.3% of patients discontinued therapy due to AEs, suggesting that this combination therapy is generally safe in hemodialysis patients [32].

#### Sofosbuvir-based therapy

The NS5B polymerase inhibitor sofosbuvir is first metabolized to the pharmacologically active nucleoside analog triphosphate GS-461203 [69], which is subsequently metabolized to the inactive metabolite GS-331007. Because GS-331007 is mainly excreted through the kidney, the GS-331007 AUC was much higher in patients with severe renal impairment (451%) or in those receiving hemodialysis (2070%) than in subjects with normal renal function. For sofosbuvir, the AUC was 60% higher in dialysis subjects than in control subjects [69]. These pharmacokinetic data indicate that exposure to the metabolite GS-331007 is dramatically increased in patients with severe renal dysfunction, which is why sofosbuvir administration is neither recommended nor contraindicated in patients with severe renal dysfunction or in those receiving hemodialysis. On the other hand, a recent study reported that, although plasma levels of GS-331007 were elevated in hemodialysis patients treated with a sofosbuvir-containing regimen compared to those in subjects with normal renal function, no accumulation of sofosbuvir or GS-331007 during the treatment was observed [70], and overexposure to GS-331007 was not associated with AE incidence.

Saxena et al. [71] reported the safety and efficacy of sofosbuvir-based therapy in patients with severe renal dysfunction registered in the HCV-TARGET database. Overall, 15 of the 17 (88%) patients with eGFR ≤ 30 mL/min/1.73 m^2^ and all 5 patients on hemodialysis at baseline achieved SVR_12_. However, compared to patients with normal renal function, those with severe renal dysfunction experienced significantly higher rates of renal function worsening and serious AEs.

On the other hand, several clinical studies have reported high efficacies (SVR rate: 88%, 7/8–100%, 15/15) and safeties of sofosbuvir-containing regimens in dialysis patients [72, 73], but those studies had only small sample sizes. A clinical trial regarding the use of sofosbuvir plus ribavirin or ledipasvir/sofosbuvir in HCV-infected patients with renal dysfunction is ongoing (NCT01958281).

#### Glecaprevir/pibrentasvir

The second-generation NS3/4A protease inhibitor glecaprevir and the second-generation NS5A inhibitor pibrentasvir do not undergo significant renal excretion. Indeed, no clinically significant increase in glecaprevir/pibrentasvir exposure was observed in patients with renal dysfunction [74]. A phase 3 trial regarding glecaprevir/pibrentasvir therapy in patients with CKD stage 4–5 included 104 HCV-infected patients (GT1, 50%; GT2, 16%; GT3, 11%; GT4, 19%; GT5-6, 2%) with severe renal dysfunction and reported an overall SVR_12_ rate of 98%, with most AEs being mild or moderate [75]. These results are very promising, suggesting that this pan-genotypic combination therapy could be used in dialysis patients with HCV GT2,3,5,6 infection, for which no approved drug was available until recently.

The approval of DAA regimens differs depending on the country or geographical area considered. When choosing the DAA regimen, the appropriate guidelines should be consulted, such as those issued by the American Association for the Study of Liver Diseases, the European Association for the Study of the Liver, or the Japan Society of Hepatology. In Japan, recommendations are based on treatment efficacy, safety, and duration; thus, paraparesis/ritonavir and elbasvir as well as grazoprevir and elbasvir are recommended for hemodialysis patients with GT1 HCV infection, while glecaprevir and pibrentasvir are expected to be effective in hemodialysis patients with HCV infection, regardless of GT.

## HCV infection in patients with HIV co-infection

Despite decreasing rates of AIDS-related mortality in HIV/HCV co-infected patients [20], most liver-disease-related deaths are thought to be due to HCV infection, liver fibrosis progresses more rapidly, and the risk of developing liver cirrhosis is high [18] [19], prompting the need for adequate HCV treatment in this patient population. We summarize previous and current anti-HCV therapy strategies for patients with HIV/HCV co-infection in Table 3.

**Table 3:** Outcomes of combination therapies with IFN-free DAAs in patients with HIV/HCV co-infection

Treatment regimen/ study	Type of study	Treatment duration (weeks)	HCV GT	Sample size	SVR rate (%)	SAE rate (%)	Treatment discontinuation rate (%)	Comments
Sofosbuvir/ribavirin								
PHOTON-1 Sulkowski et al. [84]	CT	12–24	GT1–3	223	GT1 24 W: 76% (87/114) GT2 12 W: 88% (23/26) GT2 24 W: 92% (22/24) GT3 12 W: 67% (28/42) GT3 24 W: 94% (16/17)	6% (14/223)	3% (7/223)	
PHOTON-2 Molina et al. [85]	CT	12–24	GT1–4	274	GT1 24 W: 85% (95/112) GT2 12 W: 89% (17/19) GT2 24 W: 83% (5/6) GT3 24 W: 89% (94/106) GT4 24 W: 84% (26/31)	1% (4/274)	2% (6/274)	
Sofosbuvir/ledipasvir								Co-administration of ledipasvir and TDF caused an increase in TDF serum concentration
ERADICATE study Osinusi et al. [35]	CT	12	GT1	50	98% (49/50)	2% (1/50)	0	
ION-4 Naggie et al. [36]	CT	12	GT1/4	335	96% (322/335)	2% (8/335)	0	
PTV/RTV/OBV and DSV								Should be monitored for drug–drug interactions with RTV
TURQUOISE-1 Sulkowski et al. [87]	CT	12–24	GT1	63	12 W: 94% (29/31) 24 W: 91% (29/32)	0	0	
Elbasvir/grazoprevir								
C-EDGE CO-INFECTION Rockstroh et al. [88]	CT	12	GT1/4/6	218	96% (210/218)	1% (2/218)	0	
Sofosbuvir/velpatasvir								Renal function should be monitored in TDF with velpatasvir co-administration
ASTRAL-5 Wyles et al. [107]	CT	12	GT1–4	106	95% (101/106)	2% (2/106)	2% (2/106)	
Sofosbuvir/daclatasvir								
ALLY-2 Hinestrosa et al. [90]	CT	8-12	GT1–4	203	12 W: 97.4% (149/153) 8 W: 76% (38/50)	2% (4/203)	0	

*CT* clinical trials, *RW* real-world data, *IFN* interferon, *DAAs* direct-acting antivirals, *HIV* human immunodeficiency virus, *HCV* hepatitis C virus, *12* *W* at 12 weeks, *24* *W* at 24 weeks, *GT* genotype, *SVR* sustained viral response, *SAE* serious adverse event, *TDF* tenofovir disoproxil fumarate

### IFN-based therapy in patients with HIV/HCV co-infection

Combination therapy with Peg-IFN and ribavirin used to be the standard strategy in HIV/HCV-co-infected patients. Successful eradication of HCV in such patients is believed to reduce the incidence of hepatocellular carcinoma, slow the progress to liver cirrhosis, and decrease liver-disease-related mortality [76–78]. However, the SVR rates were lower in patients with HIV/HCV co-infection (27–40%) than in those with HCV monoinfection, while the discontinuation rate due to AEs was high (12–39%) [79–81].

### Peg-IFN + ribavirin + protease inhibitor in patients with HIV/HCV co-infection

The first reports regarding the outcomes of triple therapy with Peg-IFN, ribavirin, and an HCV protease inhibitor [82, 83] indicated improved SVR rates over those of Peg-INF and ribavirin therapy in HIV/HCV-co-infected patients. However, the efficacy and safety profiles have so far been inferior to those of IFN-free DAA therapy, which is why these triple therapies have not been adopted as the standard strategy in HIV/HCV-co-infected patients.

### IFN-free DAA therapy in patients with HIV/HCV co-infection

While drug–drug interactions between DAAs and ARTs should be carefully monitored, the safety and efficacy of IFN-free DAA therapy in HIV/HCV-co-infected patients are similar to its safety and efficacy in HCV monoinfected patients.

#### Sofosbuvir and ribavirin

Two large phase 3 trials (PHOTON-1 and PHOTON-2) reported the efficacy and safety of sofosbuvir and ribavirin in patients co-infected with HCV GT1-4 and HIV [84, 85]. In PHOTON-1, 223 HIV patients co-infected with HCV/GT1-3 were assigned to receive body-weight-adjusted ribavirin and sofosbuvir combination therapy for 12 weeks (treatment-naïve HCV GT2,3) or 24 weeks (HCT GT1 and treatment-experienced HCV GT2,3). The SVR_12_ rates were 76, 88, and 67% in HIV patients with HCV GT1, treatment-naive HCV GT2, and treatment-naïve HCV GT3, respectively (12-week regimen), compared to 92 and 94% in HIV patients with treatment-experienced HCV GT2 and treatment-experienced HCV GT3, respectively (24-week regimen). Seven patients (3%) discontinued HCV treatment due to AEs. The PHOTON-2 trial reported the efficacy and safety of sofosbuvir and ribavirin in 274 HIV patients co-infected with HCV GT1-4. The SVR_12_ rates for the 24-week regimen were 85, 88, 89, and 84% in HIV patients with HCV GT1, GT2, GT3, and GT4, respectively. Six patients (2%) discontinued treatment due to AEs, and four patients (1%) experienced serious AEs.

### Sofosbuvir/ledipasvir

The efficacy and safety of sofosbuvir/ledipasvir combination therapy for both ART-naïve and ART-treated HIV/HCV-co-infected patients were reported by several clinical trials and real-world studies [35–37]. In the phase 2 ERADICATE trial, 50 treatment-naïve, noncirrhotic patients co-infected with HIV and HCV GT1 were treated with sofosbuvir/ledipasvir [35]. Overall, 98% of patients (95% CI 89–100%; 49/50) achieved SVR_12_ and no patients discontinued the treatment due to AEs. In the phase 3 ION-4 trial, 335 patients co-infected with HIV and HCV (75, 23, and 2% were GT1a, GT1b, and GT4, respectively; 20% had liver cirrhosis) receiving ART were treated with a 12-week sofosbuvir/ledipasvir regimen [36]. Overall, 96% of patients (95% CI 93–98%; 322/335) achieved SVR_12_ (96, 96, and 100% of patients with HCV GT1a, GT1b, and GT4, respectively). Among cirrhosis patients, 94% (63/67) achieved SVR_12_. All patients completed this treatment, and no lethal AEs were observed. Of the 966 HIV/HCV-co-infected patients included in the real-world study by Bhattacharya et al., 757 (78%) patients were treated with a 12-week sofosbuvir/ledipasvir ± ribavirin regimen. SVR_12_ was achieved in 92.1% (631/685) and 86.3% (113/131) of patients treated with sofosbuvir/ledipasvir and sofosbuvir/ledipasvir + ribavirin, respectively [86].

Notably, co-administration of ledipasvir and tenofovir disoproxil fumarate (TDF) as part of the ART regimen caused an increase in TDF serum concentration. Thus, when administering sofosbuvir/ledipasvir, patients receiving TDF-containing ART should be carefully monitored for AEs, including renal dysfunction.

#### Paritaprevir/ritonavir/ombitasvir/dasabuvir

The phase 3 trial TURQUOISE-1 reported the efficacy and safety of paritaprevir/ritonavir/ombitasvir/dasabuvir (3D) therapy for HIV patients co-infected with HCV GT1 and receiving ART [87]. In this study, 63 patients were treated with 12- or 24-week 3D/ribavirin regimens. The SVR rate was 94% (95% CI 79–98%; *n* = 31) for the 12-week regimen and 91% (95% CI 76–97%; *n* = 32) for the 24-week regimen. One patient withdrew consent, two experienced virological failure, and two experienced HCV re-infection. AEs were generally mild, and no patients discontinued treatment due to AEs.

#### Elbasvir/grazoprevir

The phase 3 trial C-EDGE CO-INFECTION reported the efficacy and tolerability of grazoprevir and elbasvir for HIV patients co-infected with GT1,4,6. Both ART-naïve and ART-treated HIV/HCV co-infected patients were included [88]. Overall, the SVR_12_ rate was 96% (95% CI 92.9–98.4%; 210/218). Eight patients did not achieve SVR_12_ (one due to nonvirological causes; seven experienced virological relapse). All 35 patients with liver cirrhosis achieved SVR_12_. No patients discontinued treatment due to AEs. Two patients who received ART experienced HIV viremia during treatment, but HIV plasma levels eventually became undetectable without requiring a change in the ART regimen.

#### Sofosbuvir/velpatasvir

The phase 3 trial ASTRAL-5 reported the safety and efficacy of sofosbuvir and the NS5A inhibitor velpatasvir for HIV patients who were co-infected with HCV GT1-4 and receiving ART [89]. The overall SVR_12_ rate was 95% (95% CI 89–99%; 101/106), with a SVR_12_ rate of 95% (74/78), 100% (11/11), 92% (11/12), and 100% (5/5) for patients with HCV GT1, GT2, GT3, and GT4, respectively. All 19 patients with liver cirrhosis achieved SVR_12_. Of the five patients who failed to achieve SVR_12_, two had virological failure and the other three had nonvirological failure (two were lost to follow-up and one withdrew consent). In this trial, two patients (1.9%) discontinued treatment due to AEs (one achieved SVR_12_, the other withdrew consent). Although no clinically significant change in renal function was recorded, it should be noted that no patients with creatinine clearance < 60 mL/min were included in this study. Therefore, renal function should be carefully monitored in patients receiving TDF with sofosbuvir/velpatasvir co-administration.

#### Sofosbuvir/daclatasvir

The phase III trial ALLY-2 reported the efficacy and safety of 8-week or 12-week regimens with daclatasvir plus sofosbuvir in HIV patients co-infected with HCV GT1-4 (83% with GT1) [90]. Treatment-naïve patients (*n* = 151) were randomized to receive either the 8-week or 12-week treatment, whereas all treatment-experienced patients (*n* = 52) received the 12-week treatment. The SVR_12_ rates for the 12-week treatment were 97% (95% CI 91.6–99.4%) and 98.1% (95% CI 89.7–100%) in treatment-naïve and treatment-experienced patients, respectively. On the other hand, SVR_12_ was not as high for the 8-week treatment (76.0%; 95% CI 61.8–86.9%). All patients completed the treatment, and the most common AEs were fatigue, nausea, and headache.

As explained in this section and illustrated in Table 3, IFN-free DAA combination therapy is highly effective and very well tolerated in HCV/HIV-co-infected patients, although the risk of drug–drug interactions between DAAs and ARTs should be kept in mind, and the treatment regimen should be chosen with consideration of the co-administered ARTs. It is expected that HCV protease inhibitors (asunaprevir, paritaprevir, grazoprevir) should be able to be co-administered with no significant drug–drug interactions with ARTs such as integrase inhibitors (raltegravir, dolutegravir) and some nucleoside analog reverse transcriptase inhibitors (lamivudine, TDF). Ledipasvir is known to interact with TDF, elevating the TDF blood concentration. Thus, AEs such as renal dysfunction should be monitored. When applying DAA therapy in patients with HCV/HIV co-infection, collaboration with the HIV treatment specialist should be sought. A detailed list of drug–drug interactions between DAAs and ARTs, as well as advice regarding the optimal timing for DAA therapy initiation in HIV/HCV-co-infected patients, can be found in the Department of Health and Human Services treatment guidelines (www.aidsinfo.nih.gov). Basically, ART should always be considered in patients with HIV/HCV co-infection, regardless of CD4 cell count. In ART-naïve patients with a CD4 count > 500 cells/mm^3^, ART may be deferred until the completion of anti-HCV therapy. In HCV/HIV-co-infected patients with CD4 counts of < 200 cells/mm^3^, it may be advisable to initiate ART first.

## HCV recurrence after liver transplantation

In LT recipients, recurrent HCV infection is universal and HCV replication begins within hours after LT [15], causing rapid progression of liver fibrosis [16, 17]. Within the first 5 years post-transplantation, 20–54% of patients with recurrent HCV infection develop liver cirrhosis [17], and, once liver cirrhosis is established, 42% of these patients develop decompensated liver cirrhosis within 1 year [16]. On the other hand, successful HCV eradication is significantly associated with longer survival of patients with recurrent HCV infection after LT [91]. In addition, a small number of patients with recurrent HCV infection develop fibrosing cholestatic hepatitis, which, if left untreated, leads to rapid graft loss within several months. Thus, the need for effective and safe anti-HCV therapy is especially acute in patients with recurrent HCV infection after LT.

### IFN-based therapy in patients with HCV recurrence after liver transplantation

Before the development of DAAs, anti-HCV treatment was especially challenging in patients with recurrent HCV infection after LT, as IFN-based therapy (Peg-IFN plus ribavirin) did not provide satisfactory outcomes and moreover increased the risk of liver graft rejection during the treatment [92–94]. A systematic review of 16 studies describing the outcomes of Peg-IFN/ribavirin therapy in 611 patients with recurrent HCV infection revealed an overall SVR_24_ rate of 30.2% (range 8–50%), and approximately 30% of patients discontinued the therapy due to AEs [95]. Recent reports have demonstrated the safety and efficacy of combination therapy with HCV protease inhibitor/Peg-IFN plus ribavirin in patients with recurrent HCV infection after LT [93, 96], but efficacy and discontinuation rates varied widely (SVR rate, 20–84%; discontinuation rate, 0–70%). Importantly, Levitsky et al. reported that patients with recurrent HCV infection after LT who received Peg-IFN-based therapy occasionally developed severe immune-mediated graft dysfunction, resulting in shorter graft survival [97]. Thus, in the era of IFN-free DAAs, IFN-based therapy is not recommended as the standard treatment in patients with recurrent HCV infection after LT.

### IFN-free DAA therapies in patients with HCV recurrence after liver transplantation

Several IFN-free DAA regimens have been proven to have high efficacy and safety in patients with recurrent HCV infection after LT (Table 4). However, such treatments should be applied carefully, with consideration of the potential drug–drug interactions between DDAs and immunosuppressants such as calcineurin inhibitors (cyclosporine, tacrolimus). The HCV protease inhibitors simeprevir, grazoprevir, and elbasvir are not recommended for co-administration with cyclosporine. In addition, it is necessary to monitor blood tacrolimus levels during co-administration of simeprevir or grazoprevir. Similarly, co-administration of any major immunosuppressant with paritaprevir/ritonavir/ombitasvir + dasabuvir should only be performed while carefully monitoring the serum levels of immunosuppressant [98].

**Table 4:** Outcomes of combination therapies with IFN-free DAAs in patients with recurrent HCV infection after liver transplantation in HCV/HCV co-infected patients

Treatment regimen (treatment duration) study	Type of study	Treatment duration (weeks)	HCV GT	Sample size	SVR rate (ITT)	SAE rate (%)	Treatment discontinuation rate (%)	Comments
Sofosbuvir/ribavirin								
Charlton et al. [99]	CT	24	GT1,3,4	40	70% (28/40)	15% (6/40)	5% (2/40)	
Daclatasvir/asunaprevir								
Ikegami et al. [85]	RW	24	GT 1b	74	80.3%		18%	
Sofosbuvir/ledipasvir/RBV								
SOLAR-1 Charlton et al. [38]	CT	12–24	GT1, (4)	223	CH or CP-A: 96–98% CP-B: 85–88% CP-C: 60–75%	11 to 75%	0–12%	
SOLAR-2 Manns et al. [101]	CT	12–24	GT1, 4	227	CH/CP-A: 93–100% (GT1) CP-B: 95–100% (GT1) CP-C: 50–80%	9 to 67%	3.5% (8/227)	
Sofosbuvir/ledipasvir								
Ueda et al. [102]	RW	12	GT1	54	98 (53/54)	13% (7/54)	2% (1/54)	
Sofosbuvir/daclatasvir/RBV								
ALLY-1 Poordad et al. [103]	CT	12	GT1,3,6	53	94 (50/53)	9% (5/53)	2% (1/53)	
PTV/RTV/OBV/DSV + RBV								Concentration of CI should be carefully monitored due to interactions with RTV
CORAL-2 Kwo et al. [39]	CT	24	GT1	34	96% (33/34)	6% (2/34)	3% (1/34)	
SMV/sofosbuvir ± RBV								
Pungpapong et al. [105]	RW	12	GT1	123	90% (94/105)	2% (3/123)	2% (3/123)	
Brown et al. [106]	RW	12–24	GT1	151	88% (133/151)	12% (18/151)		

*CT* clinical trials, *RW* real-world data, *IFN* interferon, *DAAs* direct-acting antivirals, *HCV* hepatitis C virus, *GT* genotype, *SVR* sustained viral response, *SAE* serious adverse event, *CP* Child–Pugh classification (classes A through C), *ITT* intention to treat, *CH* chronic hepatitis, *RBV* ribavirin, *SMV* simeprevir, *RTV* ritonavir, *CI* calcineurin inhibitor

#### Sofosbuvir/ribavirin

The first report on IFN-free DAA therapy in patients with recurrent HCV infection after LT described the outcomes of a 24-week sofosbuvir/ribavirin regimen. Of the 40 patients enrolled, 83% were infected with HCV GT1 (the rest were infected with HCV GT3,4) and 40% had liver cirrhosis. The overall SVR_12_ rate was 70% (90% CI 56–82%; 28/40). Two patients (5%) discontinued this combination therapy due to AEs, but no patients experienced death, lethal AEs, graft loss, or graft rejection episodes [99].

#### Daclatasvir + asunaprevir

Recently, Ikegami et al. reported the safety and efficacy of a 24-week regimen with asunaprevir and daclatasvir but without ribavirin or IFN in a multicenter study enrolling 74 Japanese patients with recurrent HCV GT1b infection after LT. The overall SVR rate was 80.3%. Previous history of simprevir treatment and infection with HCV with NS5A resistance-associated variants at baseline were significantly associated with non-SVR. The majority of the patients (82.4%) completed this treatment, and no lethal AEs or deaths were noted [100].

#### Ledipasvir/sofosbuvir with or without ribavirin

The large randomized phase 2 study SOLAR-1 reported the safety and efficacy of sofosbuvir/ledipasvir plus ribavirin treatment in 223 patients with recurrent HCV (mostly GT1) infection after LT, who were randomized to receive the 12-week or 24-week regimen. The SVR_12_ rate in patients without liver cirrhosis (*n* = 111) or with Child–Pugh grade A (*n* = 51) was 96–98%, compared to 85–88% in patients with Child–Pugh grade B and 60–75% in those with Child–Pugh grade C. The SVR rates were similar between the groups (i.e., 12-week versus 24-week regimen). The rate of treatment discontinuation due to AEs was 2%, and four patients died due to liver decompensation [38].

Similarly, the multicenter, open-label, randomized, phase 2 trial SOLAR-2, conducted in Europe, reported high efficacy and safety for sofosbuvir/ledipasvir therapy in 227 patients with HCV recurrence after LT [101]. Among patients without cirrhosis or with Child–Pugh grade A after LT, the SVR_12_ rate was 95.2% and 98.8% in the 12-week and 24-week treatment groups, respectively. Among patients with Child–Pugh grade B or C, the SVR12 rates were 33–100%. The rate of treatment discontinuation due to AEs was 2% and 7 patients died, mainly from complications related to hepatic decompensation and infection.

A very recent report regarding the efficacy and safety of a 12-week sofosbuvir/ledipasvir regimen without ribavirin described 54 Japanese patients with recurrent HCV infection (GT1b) after LT. Patients with decompensated liver cirrhosis or severe renal dysfunction were excluded from this study. The overall SVR_12_ rate was 98% (53/54). One patient developed pneumonia at 4 weeks after the initiation of treatment and died thereafter, but the remaining 53 patients completed the treatment. This sofosbuvir/ledipasvir combination treatment was well tolerated in most patients. One patient died due to pneumonia [102].

#### Daclatasvir + sofosbuvir with or without ribavirin

The phase III trial ALLY-1 demonstrated the efficacy and safety of a 12-week daclatasvir/sofosbuvir regimen ± ribavirin in 53 patients with recurrent HCV infection (GT1, *n* = 41; GT3, *n* = 11; GT6, *n* = 1) after LT. The overall SVR rate was 94% (50/53), with an SVR rate of 97% for GT1a, 90% for GT1b, and 91% for GT3. One patient (2%) discontinued treatment due to AEs, five patients (9%) experienced serious AEs, but no deaths were noted [103].

Fontana et al. reported the results of regimens involving sofosbuvir/daclatasvir ± ribavirin or daclatasvir/simprevir plus ribavirin in patients with recurrent HCV infection after LT. In this study, 77 patients were treated with sofosbuvir/daclatasvir and ribavirin, among whom the SVR rate was 91% [104].

#### Paritaprevir/ritonavir/ombitasvir/dasabuvir + ribavirin

The phase 2 trial CORAL-II reported the efficacy and safety of a 24-week regimen involving paritaprevir, ritonavir, ombitasvir, dasabuvir, and ribavirin in patients with recurrent HCV infection after LT. Thirty-four patients with HCV GT1 recurrence after LT and mild liver fibrosis (F0-F2) were included in this multicenter study. The overall SVR_24_ rate was 97% (95% CI 85–100%). One patient discontinued this therapy due to AEs, and two patients experienced serious AEs, but no death was noted. Because the regimen contained ritonavir, the blood concentration of calcineurin inhibitors was carefully monitored [39].

#### Simeprevir + sofosbuvir

Several studies demonstrated the safety and efficacy of combination therapy with sofosbuvir/simeprevir ± ribavirin. Pungpapong et al. conducted a retrospective multicenter study that enrolled 123 patients with HCV GT1 recurrence after LT. The overall SVR_12_ rate was 90% [105]. Three patients discontinued this therapy due to AEs, and one patient died due to pneumonitis. Brown et al. reported the outcomes of a 12-week or 24-week sofosbuvir/simprevir regimen ± ribavirin in patients with recurrent HCV infection after LT (HCV-TARGET study) [106]. This study included 151 patients with recurrent HCV GT1 infection, and 64.2% of patients had liver cirrhosis. The overall SVR rate was 88% (95% CI 82–93%; 133/151), with 11.9% of patients experiencing serious AEs and 4 patients discontinuing these therapies due to AEs. Three patients died due to pneumonia, suicide, and multi-organ failure.

The approval of DAA regimens differs with the country or geographical area. In Japan, recommendations for HCV treatment in LT recipients take into consideration real-world data; specifically, sofosbuvir and ledipasvir are recommended for patients with recurrent HCV GT1 infection after LT.

## Conclusion

In this review, we discussed the efficacy and safety of novel DAAs in special populations consisting of hemodialysis patients, patients with HIV/HCV co-infection, and patients with recurrent HCV infection after LT. The development of novel DAAs has dramatically changed the landscape of anti-HCV therapy in these special populations, which were historically classified as difficult to treat. The SVR rate of IFN-free DAA therapies in such patients is very high (> 90%), with fewer and less-severe AEs. Until recently, no IFN-free DAA regimen had been approved for hemodialysis patients with HCV GT2,3,5,6 in any country. However, the clinical trial of glecaprevir and pibrentasvir revealed that this combination therapy might address this unmet need in hemodialysis patients. However, several issues remain. The data on sofosbuvir treatment outcomes in dialysis patients are limited, so the use of sofosbuvir in dialysis patients remains off-label and should only be conducted by experienced physicians with the full informed consent of the patients. In addition, considering the risk of drug–drug interactions between DAAs and ARTs in patients with HIV/HCV co-infection, or between DAAs and immunosuppressive drugs in patients with recurrent HCV infection after LT, careful monitoring for AEs should be performed, and further data on such interactions should be obtained. We are convinced that such aspects will be clarified in the near future based on further data from ongoing clinical trials and real-world studies.

## References

[1] Gower E, Estes C, Blach S (2014). Global epidemiology and genotype distribution of the hepatitis C virus infection. J Hepatol.

[2] Mohd Hanafiah K, Groeger J, Flaxman AD (2013). Global epidemiology of hepatitis C virus infection: new estimates of age-specific antibody to HCV seroprevalence. Hepatology.

[3] Seeff LB (2002). Natural history of chronic hepatitis C. Hepatology.

[4] Kim WR, Stock PG, Smith JM, et al. OPTN/SRTR 2011 annual data report: liver. Am J Transpl. 2013;13(Suppl 1):73–102 **Epub 2013/01/31**. 10.1111/ajt.12021 23237697

[5] Gill K, Ghazinian H, Manch R (2016). Hepatitis C virus as a systemic disease: reaching beyond the liver. Hepatol Int.

[6] Kidney Disease: Improving Global Outcomes. KDIGO clinical practice guidelines for the prevention, diagnosis, evaluation, and treatment of hepatitis C in chronic kidney disease. Kidney Int Suppl. 2008(109):S1–S99 10.1038/ki.2008.81 18382440

[7] Iwasa Y, Otsubo S, Sugi O (2008). Patterns in the prevalence of hepatitis C virus infection at the start of hemodialysis in Japan. Clin Exp Nephrol.

[8] Di Napoli A, Pezzotti P, Di Lallo D (2006). Epidemiology of hepatitis C virus among long-term dialysis patients: a 9-year study in an Italian region. Am J Kidney Dis.

[9] Fissell RB, Bragg-Gresham JL, Woods JD (2004). Patterns of hepatitis C prevalence and seroconversion in hemodialysis units from three continents: the DOPPS. Kidney Int.

[10] Sherman KE, Rouster SD, Chung RT, et al. Hepatitis C virus prevalence among patients infected with human immunodeficiency virus: a cross-sectional analysis of the US adult AIDS Clinical Trials Group. Clin Infect Dis. 2002;34(6):831–7. 10.1086/339042 11833007

[11] Thomas DL (2008). The challenge of hepatitis C in the HIV-infected person. Annu Rev Med.

[12] Nakayama E, Akiba T, Marumo F (2000). Prognosis of anti-hepatitis C virus antibody-positive patients on regular hemodialysis therapy. J Am Soc Nephrol.

[13] Goodkin DA, Bragg-Gresham JL, Koenig KG (2003). Association of comorbid conditions and mortality in hemodialysis patients in Europe, Japan, and the United States: the Dialysis Outcomes and Practice Patterns Study (DOPPS). J Am Soc Nephrol.

[14] Mathurin P, Mouquet C, Poynard T (1999). Impact of hepatitis B and C virus on kidney transplantation outcome. Hepatology.

[15] Garcia-Retortillo M, Forns X, Feliu A (2002). Hepatitis C virus kinetics during and immediately after liver transplantation. Hepatology.

[16] Berenguer M, Prieto M, Rayon JM (2000). Natural history of clinically compensated hepatitis C virus-related graft cirrhosis after liver transplantation. Hepatology.

[17] Berenguer M, Schuppan D (2013). Progression of liver fibrosis in post-transplant hepatitis C: mechanisms, assessment and treatment. J Hepatol.

[18] Benhamou Y, Bochet M, Di Martino V (1999). Liver fibrosis progression in human immunodeficiency virus and hepatitis C virus coinfected patients. The Multivirc Group. Hepatology.

[19] Graham CS, Baden LR, Yu E (2001). Influence of human immunodeficiency virus infection on the course of hepatitis C virus infection: a meta-analysis. Clin Infect Dis.

[20] Smith CJ, Ryom L, Weber R (2014). Trends in underlying causes of death in people with HIV from 1999 to 2011 (D:A:D): a multicohort collaboration. Lancet.

[21] Gordon CE, Uhlig K, Lau J (2008). Interferon treatment in hemodialysis patients with chronic hepatitis C virus infection: a systematic review of the literature and meta-analysis of treatment efficacy and harms. Am J Kidney Dis.

[22] Ayaz C, Celen MK, Yuce UN (2008). Efficacy and safety of pegylated-interferon alpha-2a in hemodialysis patients with chronic hepatitis C. World J Gastroenterol.

[23] Brau N, Rodriguez-Torres M, Prokupek D (2004). Treatment of chronic hepatitis C in HIV/HCV-coinfection with interferon alpha-2b + full-course vs. 16-week delayed ribavirin. Hepatology.

[24] Omata M, Nishiguchi S, Ueno Y (2014). Sofosbuvir plus ribavirin in Japanese patients with chronic genotype 2 HCV infection: an open-label, phase 3 trial. J Viral Hepat.

[25] Feld JJ, Kowdley KV, Coakley E (2014). Treatment of HCV with ABT-450/r-ombitasvir and dasabuvir with ribavirin. N Engl J Med.

[26] Kowdley KV, Gordon SC, Reddy KR (2014). Ledipasvir and sofosbuvir for 8 or 12 weeks for chronic HCV without cirrhosis. N Engl J Med.

[27] Kumada H, Suzuki Y, Ikeda K (2014). Daclatasvir plus asunaprevir for chronic HCV genotype 1b infection. Hepatology.

[28] Kumada H, Chayama K, Rodrigues-Jr L, et al. Randomized phase 3 trial of ombitasvir/paritaprevir/ritonavir for HCV genotype 1b-infected Japanese patients with or without cirrhosis. Hepatology. 2015;62(4):1037–46. 10.1002/hep.27972 PMC5049673 26147154

[29] Mizokami M, Yokosuka O, Takehara T (2015). Ledipasvir and sofosbuvir fixed-dose combination with and without ribavirin for 12 weeks in treatment-naive and previously treated Japanese patients with genotype 1 hepatitis C: an open-label, randomised, phase 3 trial. Lancet Infect Dis.

[30] Suda G, Nagasaka A, Yamamoto Y, et al. Safety and efficacy of daclatasvir and asunaprevir in hepatitis C virus-infected patients with renal impairment. Hepatol Res. 2017;47(11):1127–36. 10.1111/hepr.12851 27943523

[31] Pockros PJ, Reddy KR, Mantry PS, et al. Efficacy of direct-acting antiviral combination for patients with hepatitis C virus genotype 1 infection and severe renal impairment or end-stage renal disease. Gastroenterology. 2016;150(7):1590–8. 10.1053/j.gastro.2016.02.078 26976799

[32] Suda G, Furusyo N, Toyoda H, et al. Daclatasvir and asunaprevir in hemodialysis patients with hepatitis C virus infection: a nationwide retrospective study in Japan. J Gastroenterol. 2017;53(1):119–28. 10.1007/s00535-017-1353-y 28560477

[33] Suda G, Kudo M, Nagasaka A (2016). Efficacy and safety of daclatasvir and asunaprevir combination therapy in chronic hemodialysis patients with chronic hepatitis C. J Gastroenterol.

[34] Suda G, Ogawa K, Kimura M, et al. Novel treatment of hepatitis C virus infection for patients with renal impairment. J Clin Transl Hepatol. 2016;4(4):320–7. 10.14218/JCTH.2016.00032 PMC5225152 28097101

[35] Osinusi A, Townsend K, Kohli A (2015). Virologic response following combined ledipasvir and sofosbuvir administration in patients with HCV genotype 1 and HIV co-infection. JAMA.

[36] Naggie S, Cooper C, Saag M (2015). Ledipasvir and sofosbuvir for HCV in patients coinfected with HIV-1. N Engl J Med.

[37] Sogni P, Gilbert C, Lacombe K, et al. All-oral direct-acting antiviral regimens in HIV/hepatitis C virus-coinfected patients with cirrhosis are efficient and safe: real-life results from the prospective ANRS CO13-HEPAVIH cohort. Clin Infect Dis. 2016;63(6):763–70. 10.1093/cid/ciw379 27317796

[38] Charlton M, Everson GT, Flamm SL (2015). Ledipasvir and sofosbuvir plus ribavirin for treatment of HCV infection in patients with advanced liver disease. Gastroenterology.

[39] Kwo PY, Mantry PS, Coakley E (2014). An interferon-free antiviral regimen for HCV after liver transplantation. N Engl J Med.

[40] Bergman S, Accortt N, Turner A (2005). Hepatitis C infection is acquired pre-ESRD. Am J Kidney Dis.

[41] Alavian SM, Kabir A, Ahmadi AB (2010). Hepatitis C infection in hemodialysis patients in Iran: a systematic review. Hemodial Int.

[42] Fabrizi F, Plaisier E, Saadoun D (2013). Hepatitis C virus infection, mixed cryoglobulinemia, and kidney disease. Am J Kidney Dis.

[43] Lee JJ, Lin MY, Chang JS (2014). Hepatitis C virus infection increases risk of developing end-stage renal disease using competing risk analysis. PLoS One.

[44] Kalantar-Zadeh K, Kilpatrick RD, McAllister CJ (2007). Hepatitis C virus and death risk in hemodialysis patients. J Am Soc Nephrol.

[45] Fabrizi F, Takkouche B, Lunghi G (2007). The impact of hepatitis C virus infection on survival in dialysis patients: meta-analysis of observational studies. J Viral Hepat.

[46] Akiba T, Hora K, Imawari M (2012). 2011 Japanese Society for Dialysis Therapy guidelines for the treatment of hepatitis C virus infection in dialysis patients. Ther Apher Dial.

[47] Roth D, Nelson DR, Bruchfeld A (2015). Grazoprevir plus elbasvir in treatment-naive and treatment-experienced patients with hepatitis C virus genotype 1 infection and stage 4–5 chronic kidney disease (the C-SURFER study): a combination phase 3 study. Lancet.

[48] Merck & Co. Prescribing information for Rebetol^®^ (ribavirin USP) capsules, for oral use. Kenilworth: Merck & Co.; 2016.

[49] Fabrizi F, Dixit V, Messa P (2015). Pegylated interferon mono-therapy of chronic hepatitis C in the dialysis population: systematic review and meta-analysis. Ther Apher Dial.

[50] Kumada H, Toyota J, Okanoue T (2012). Telaprevir with peginterferon and ribavirin for treatment-naive patients chronically infected with HCV of genotype 1 in Japan. J Hepatol.

[51] Poordad F, McCone J, Bacon BR (2011). Boceprevir for untreated chronic HCV genotype 1 infection. N Engl J Med.

[52] Bacon BR, Gordon SC, Lawitz E (2011). Boceprevir for previously treated chronic HCV genotype 1 infection. N Engl J Med.

[53] Suda G, Yamamoto Y, Nagasaka A (2015). Serum granulysin levels as a predictor of serious telaprevir-induced dermatological reactions. Hepatol Res.

[54] Dumortier J, Guillaud O, Gagnieu MC (2013). Anti-viral triple therapy with telaprevir in haemodialysed HCV patients: is it feasible?. J Clin Virol.

[55] Wiegand J, Maasoumy B, Buggisch P, et al. Letter: Telaprevir triple therapy in chronic hepatitis C genotype 1 patients receiving haemodialysis. Aliment Pharmacol Ther. 2014;39(11):1342–4. 10.1111/apt.12748 24803258

[56] Knapstein J, Galle PR, Zimmermann T (2014). Antiviral triple therapy with boceprevir in a chronic hepatitis C haemodialysis patient awaiting kidney re-transplantation. Dig Liver Dis.

[57] Merck & Co. US prescribing information for Zepatier™ (elbasvir and grazoprevir) tablets, for oral use. Kenilworth: Merck & Co.; 2016.

[58] Bruchfeld A, Roth D, Martin P (2017). Elbasvir plus grazoprevir in patients with hepatitis C virus infection and stage 4–5 chronic kidney disease: clinical, virological, and health-related quality-of-life outcomes from a phase 3, multicentre, randomised, double-blind, placebo-controlled trial. Lancet Gastroenterol Hepatol.

[59] AbbVie Inc. Prescribing information for VIEKIRA PAK (ombitasvir, paritaprevir, and ritonavir tablets; dasabuvir tablets), co-packaged for oral use. North Chicago: AbbVie Inc.; 2016.

[60] Atsukawa M, Tsubota A, Koushima Y, et al. Efficacy and safety of ombitasvir/paritaprevir/ritonavir in dialysis patients with genotype 1b chronic hepatitis C. Hepatol Res. 2017;47(13):1429–37. 10.1111/hepr.12910 28457003

[61] Munoz-Gomez R, Rincon D, Ahumada A (2017). Therapy with ombitasvir/paritaprevir/ritonavir plus dasabuvir is effective and safe for the treatment of genotypes 1 and 4 hepatitis C virus (HCV) infection in patients with severe renal impairment: a multicentre experience. J Viral Hepat.

[62] Bristol-Myers Squibb Co. Japanese prescribing information for Sunvepra capsules (asunaprevir). New Brunswick: Bristol-Myers Squibb Co.; 2014.

[63] Bristol-Myers Squibb Co. Japanese prescribing information for Daklinza tablets (daclatasvir). New Brunswick: Bristol-Myers Squibb Co.; 2014.

[64] Manns M, Pol S, Jacobson IM (2014). All-oral daclatasvir plus asunaprevir for hepatitis C virus genotype 1b: a multinational, phase 3, multicohort study. Lancet.

[65] Suda G, Nagasaka A, Yamamoto Y, et al. Safety and efficacy of daclatasvir and asunaprevir in hepatitis C virus infected patients with renal impairment. Hepatol Res. 2017;47(11):1127–36. 10.1111/hepr.12851 27943523

[66] Toyoda H, Kumada T, Tada T (2016). Safety and efficacy of dual direct-acting antiviral therapy (daclatasvir and asunaprevir) for chronic hepatitis C virus genotype 1 infection in patients on hemodialysis. J Gastroenterol.

[67] Kawakami Y, Imamura M, Ikeda H, et al. Pharmacokinetics, efficacy and safety of daclatasvir plus asunaprevir in dialysis patients with chronic hepatitis C: pilot study. J Viral Hepat. 2016;23(11):850–6. 10.1111/jvh.12553 27346670

[68] Miyazaki R, Miyagi K. Effect and safety of daclatasvir-asunaprevir combination therapy for chronic hepatitis C virus genotype 1b-infected patients on hemodialysis. Ther Apher Dial. 2016;20(5):462–7. 10.1111/1744-9987.12407 27098678

[69] Gilead Sciences, Inc. Prescribing information for Harvoni combination tablets^®^ (sofosbuvir + ledipasvir), for oral use. Foster City: Gilead Sciences, Inc.; 2015.

[70] Desnoyer A, Pospai D, Le MP (2016). Pharmacokinetics, safety and efficacy of a full dose sofosbuvir-based regimen given daily in hemodialysis patients with chronic hepatitis C. J Hepatol.

[71] Saxena V, Koraishy FM, Sise ME (2016). Safety and efficacy of sofosbuvir-containing regimens in hepatitis C-infected patients with impaired renal function. Liver Int.

[72] Nazario HE, Ndungu M, Modi AA (2016). Sofosbuvir and simeprevir in hepatitis C genotype 1-patients with end-stage renal disease on haemodialysis or GFR < 30 ml/min. Liver Int.

[73] Singh T, Guirguis J, Anthony S (2016). Sofosbuvir-based treatment is safe and effective in patients with chronic hepatitis C infection and end stage renal disease: a case series. Liver Int.

[74] Kosloski MDS, Zhao W. Pharmacokinetics, safety, and tolerability of next generation direct acting antivirals ABT-493 and ABT-530 in subjects with renal impairment. J Hepatol. 2016;64(2):S405–6.

[75] Gane EJ, Lawitz E, Pugatch D, et al. EXPEDITION-IV: safety and efficacy of GLE/PIB in adults with renal impairment and chronic hepatitis C virus genotype 1–6 infection. Hepatology 2016;64(6):1125A.

[76] Berenguer J, Alvarez-Pellicer J, Martin PM (2009). Sustained virological response to interferon plus ribavirin reduces liver-related complications and mortality in patients coinfected with human immunodeficiency virus and hepatitis C virus. Hepatology.

[77] Limketkai BN, Mehta SH, Sutcliffe CG (2012). Relationship of liver disease stage and antiviral therapy with liver-related events and death in adults coinfected with HIV/HCV. JAMA.

[78] Mira JA, Rivero-Juarez A, Lopez-Cortes LF (2013). Benefits from sustained virologic response to pegylated interferon plus ribavirin in HIV/hepatitis C virus-coinfected patients with compensated cirrhosis. Clin Infect Dis.

[79] Chung RT, Andersen J, Volberding P, et al. Peginterferon Alfa-2a plus ribavirin versus interferon alfa-2a plus ribavirin for chronic hepatitis C in HIV-coinfected persons. N Engl J Med. 2004;351(5):451–9. 10.1056/NEJMoa032653 PMC4113392 15282352

[80] Torriani FJ, Rodriguez-Torres M, Rockstroh JK (2004). Peginterferon Alfa-2a plus ribavirin for chronic hepatitis C virus infection in HIV-infected patients. N Engl J Med.

[81] Carrat F, Bani-Sadr F, Pol S (2004). Pegylated interferon alfa-2b vs standard interferon alfa-2b, plus ribavirin, for chronic hepatitis C in HIV-infected patients: a randomized controlled trial. JAMA.

[82] Dieterich D, Rockstroh JK, Orkin C (2014). Simeprevir (TMC435) with pegylated interferon/ribavirin in patients coinfected with HCV genotype 1 and HIV-1: a phase 3 study. Clin Infect Dis.

[83] Neukam K, Munteanu DI, Rivero-Juarez A (2015). Boceprevir or telaprevir based triple therapy against chronic hepatitis C in HIV coinfection: real-life safety and efficacy. PLoS One.

[84] Sulkowski MS, Naggie S, Lalezari J (2014). Sofosbuvir and ribavirin for hepatitis C in patients with HIV coinfection. JAMA.

[85] Molina JM, Orkin C, Iser DM (2015). Sofosbuvir plus ribavirin for treatment of hepatitis C virus in patients co-infected with HIV (PHOTON-2): a multicentre, open-label, non-randomised, phase 3 study. Lancet.

[86] Bhattacharya D, Belperio PS, Shahoumian TA, et al. Effectiveness of all-oral antiviral regimens in 996 human immunodeficiency virus/hepatitis C virus genotype 1-coinfected patients treated in routine practice. Clin Infect Dis. 2017;64(12):1711–20. 10.1093/cid/cix111 28199525

[87] Sulkowski MS, Eron JJ, Wyles D (2015). Ombitasvir, paritaprevir co-dosed with ritonavir, dasabuvir, and ribavirin for hepatitis C in patients co-infected with HIV-1: a randomized trial. JAMA.

[88] Rockstroh JK, Nelson M, Katlama C (2015). Efficacy and safety of grazoprevir (MK-5172) and elbasvir (MK-8742) in patients with hepatitis C virus and HIV co-infection (C-EDGE CO-INFECTION): a non-randomised, open-label trial. Lancet HIV.

[89] Wyles D, Brau N, Kottilil S, et al. Sofosbuvir and velpatasvir for the treatment of hepatitis C virus in patients coinfected with human immunodeficiency virus type 1: an open-label, phase 3 study. Clin Infect Dis. 2017;65(1):6–12. 10.1093/cid/cix260 PMC6248627 28369210

[90] Wyles DL, Ruane PJ, Sulkowski MS, et al. Daclatasvir plus sofosbuvir for HCV in patients coinfected with HIV-1. N Engl J Med. 2015;373(8):714–25. 10.1056/NEJMoa1503153 26196502

[91] Akamatsu N, Sugawara Y, Kokudo N (2014). Outcomes of living donor liver transplantation for hepatitis C virus-positive recipients in Japan: results of a nationwide survey. Transpl Int.

[92] Bunchorntavakul C, Reddy KR (2014). Management of hepatitis C before and after liver transplantation in the era of rapidly evolving therapeutic advances. J Clin Transl Hepatol.

[93] Coilly A, Roche B, Dumortier J (2014). Safety and efficacy of protease inhibitors to treat hepatitis C after liver transplantation: a multicenter experience. J Hepatol.

[94] Watt K, Veldt B, Charlton M. A practical guide to the management of HCV infection following liver transplantation. Am J Transpl. 2009;9(8):1707–13. 10.1111/j.1600-6143.2009.02702.x 19538491

[95] Berenguer M (2008). Systematic review of the treatment of established recurrent hepatitis C with pegylated interferon in combination with ribavirin. J Hepatol.

[96] Ikegami T, Yoshizumi T, Kato M (2014). Reduced-dose telaprevir-based triple antiviral therapy for recurrent hepatitis C after living donor liver transplantation. Transplantation.

[97] Levitsky J, Fiel MI, Norvell JP (2012). Risk for immune-mediated graft dysfunction in liver transplant recipients with recurrent HCV infection treated with pegylated interferon. Gastroenterology.

[98] Belli LS, Duvoux C, Berenguer M (2017). ELITA consensus statements on the use of DAAs in liver transplant candidates and recipients. J Hepatol.

[99] Charlton M, Gane E, Manns MP (2015). Sofosbuvir and ribavirin for treatment of compensated recurrent hepatitis C virus infection after liver transplantation. Gastroenterology.

[100] Ikegami T, Ueda Y, Akamatsu N, et al. Asunaprevir and daclatasvir for recurrent hepatitis C after liver transplantation: a Japanese multicenter experience. Clin Transplant. 2017;31(11):e13109. 10.1111/ctr.13109 28881052

[101] Manns M, Samuel D, Gane EJ (2016). Ledipasvir and sofosbuvir plus ribavirin in patients with genotype 1 or 4 hepatitis C virus infection and advanced liver disease: a multicentre, open-label, randomised, phase 2 trial. Lancet Infect Dis.

[102] Ueda Y, Ikegami T, Akamatsu N (2017). Treatment with sofosbuvir and ledipasvir without ribavirin for 12 weeks is highly effective for recurrent hepatitis C virus genotype 1b infection after living donor liver transplantation: a Japanese multicenter experience. J Gastroenterol.

[103] Poordad F, Schiff ER, Vierling JM (2016). Daclatasvir with sofosbuvir and ribavirin for hepatitis C virus infection with advanced cirrhosis or post-liver transplantation recurrence. Hepatology.

[104] Fontana RJ, Brown RS, Moreno-Zamora A (2016). Daclatasvir combined with sofosbuvir or simeprevir in liver transplant recipients with severe recurrent hepatitis C infection. Liver Transpl.

[105] Pungpapong S, Aqel B, Leise M (2015). Multicenter experience using simeprevir and sofosbuvir with or without ribavirin to treat hepatitis C genotype 1 after liver transplant. Hepatology.

[106] Brown RS, O’Leary JG, Reddy KR (2016). Interferon-free therapy for genotype 1 hepatitis C in liver transplant recipients: real-world experience from the hepatitis C therapeutic registry and research network. Liver Transpl.

[107] Younossi ZM, Stepanova M, Sulkowski M, et al. Patient-reported outcomes in patients co-infected with hepatitis C virus and human immunodeficiency virus treated with sofosbuvir and velpatasvir: the ASTRAL-5 study. Liver Int. 2017;37:1796–804. 10.1111/liv.13462 28470938

